# The Effect of Rumination Time on Milk Performance and Methane Emission of Dairy Cows Fed Partial Mixed Ration Based on Maize Silage

**DOI:** 10.3390/ani12010050

**Published:** 2021-12-27

**Authors:** Robert Mikuła, Marcin Pszczola, Katarzyna Rzewuska, Sebastian Mucha, Włodzimierz Nowak, Tomasz Strabel

**Affiliations:** 1 Department of Animal Nutrition, Poznań University of Life Sciences, Wołyńska 33, 60-637 Poznan, Poland; wlodzimierz.nowak@up.poznan.pl; 2 Department of Genetics and Animal Breeding, Poznań University of Life Sciences, Wołyńska 33, 60-637 Poznan, Poland; katarzyna.rzewuska@up.poznan.pl (K.R.); sebastian.mucha@up.poznan.pl (S.M.); tomasz.strabel@up.poznan.pl (T.S.)

**Keywords:** rumination, chewing activity, milk production, methane emission, automatic milking

## Abstract

**Simple Summary:**

Greenhouse gas emission has attracted considerable public attention in recent years, driving the search for genetic, nutritional, and management strategies to reduce methane emissions and increase the sustainability of milk production. Rumination activity has an important function in feed particle size reduction, condition of feeding behavior, and feed intake as well as in stabilizing rumen fluid pH through saliva production. A total of 365 high-yielding Polish Holstein -Friesian multiparous dairy cows were included in the study covering 24 to 304 days of lactation. Next, the data from the cows were assigned to three groups based on daily rumination time: low rumination up to 412 min/day (up to 25th rumination percentile), medium rumination from 412 to 527 min/day (between the 25th and 75th percentile), and high rumination above 527 min/day (from the 75th percentile). We showed that a longer rumination time leads to a lower methane emission level. Therefore, strategies that increase chewing activity may be used to reduce the environmental impact of dairy cows production.

**Abstract:**

The objective of this study was to determine the effect of the rumination time on milk yield and composition as well as methane emission during lactation in high-yielding dairy cows fed a partial mixed ration based on maize silage without pasture access. A total of 365 high-yielding Polish Holstein-Friesian multiparous dairy cows were included in the study covering 24 to 304 days of lactation. Methane emission, rumination time, and milk production traits were observed for the period of 12 months. Next, the data from the cows were assigned to three groups based on daily rumination time: low rumination up to 412 min/day (up to 25th rumination percentile), medium rumination from 412 to 527 min/day (between the 25th and 75th percentile), and high rumination above 527 min/day (from the 75th percentile). Rumination time had no effect on milk yield, energy-corrected milk yield, or fat and protein-corrected milk yield. High rumination time had an effect on lower fat concentration in milk compared with the medium and low rumination groups. The highest daily CH_4_ production was noted in low rumination cows, which emitted 1.8% more CH_4_ than medium rumination cows and 4.2% more than high rumination cows. Rumination time affected daily methane production per kg of milk. Cows from the high rumination group produced 2.9% less CH_4_ per milk unit compared to medium rumination cows and 4.6% in comparison to low rumination cows. Similar observations were noted for daily CH_4_ production per ECM unit. In conclusion, a longer rumination time is connected with lower methane emission as well as lower methane production per milk unit in high-yielding dairy cows fed a maize silage-based partial mixed ration without pasture access.

## 1. Introduction

The milk production of dairy cows has increased substantially over the last few years due to selection as well as substantially improved nutrition and herd management. High production requires substituting forage with a high starch content concentrate to meet the high nutrient requirement as well as maintain rumen homeostasis. As a consequence, the contribution of crude fiber and physically effective neutral detergent fiber to the diet of high-yielding dairy cows has decreased. In turn, this can affect the rumination behavior. Rumination is desirable, as it takes part in breaking down of the feed particles and stimulates saliva production. Saliva lysozyme through preventing the proliferation of Gram-positive bacteria plays an important function on the rumen microbiota and can also influence the selection of methanogenic microorganisms and affect the rumen ecosystem and modulate methane emissions. Saliva also contains bicarbonate and phosphate buffers and plays an important role in sustaining the rumen fluid pH and cellulolytic microbial activity [1]. Thus, the optimal rumination activity is necessary to decrease the risk of rumen subacute and acute acidosis [2,3] as well as maintain good health status and lower incidences of clinical and subclinical disorders [4,5,6,7]. Rumination impacts the whole digestion process, including the feed passage rate as well as voluntary feed intake in dairy cows [8], while it may impact the cow’s milk performance [9]. Watt et al. [10] showed that a longer rumination time improves feed intake, milk production, and total methane emission, while it also reduces methane emission per milk unit during the 22-day experimental period in grazing dairy cows.

Greenhouse gas emission by dairy farms has become the focus of public attention in recent years. The search for nutritional and management methods to reduce methane emission is necessary for sustainable milk production [11,12]. The rumen environment may affect methane synthesis by the rumen methanogens [13]. An increase in acetate and butyrate contents in the rumen fluid can affect the concentration of dissolved hydrogen utilized in methane synthesis [14]. The rise of acetate fermentation is related to the availability of crude fiber and creates a homeostatic environment related to fiber degradation bacteria [1]. As described above, rumination time due to its role in stabilizing pH of rumen fluid is related to the health status of cows and also can indirectly affect the rise of methane emission. In the available literature, the relationships between both phenotypes—rumination time and methane emission—has been described mainly in grazing dairy cows [10]. Despite other studies, which mainly focused on the description of genetics correlations between rumination time and methane emission, there is a lack of a study covering high-yielding dairy cows fed a diet based on maize silage during the whole lactation period. Additionally, results of the published experiments covered only a small part of lactation [10,15] or were conducted on other than Polish Holstein-Friesian breed [16] or aimed to compare different methods of methane measurement [17], whereas the present study analyzed records from 24 to 304 days of lactation on 365 animals to provide a better overview of interactions between rumination activity, performance, and methane production.

We hypothesized that a longer rumination time is connected with lower methane emission per milk unit in high yielding dairy cows fed without pasture access.

The objective of this study was to determine the effect of the rumination time, milk yield, and composition along with methane emission during lactation in high-yielding dairy cows fed a maize silage-based partial mixed ration.

## 2. Materials and Methods

### 2.1. Animal Management, Experimental Design, and Diet

All animal procedures were conducted in accordance with the guidelines of the Polish Council for Animal Care and the Local Ethics Commission of the Poznań University of Life Sciences (Poznań, Poland) with respect to animal experimentation and care of the animals under the study.

**Figure 1:**
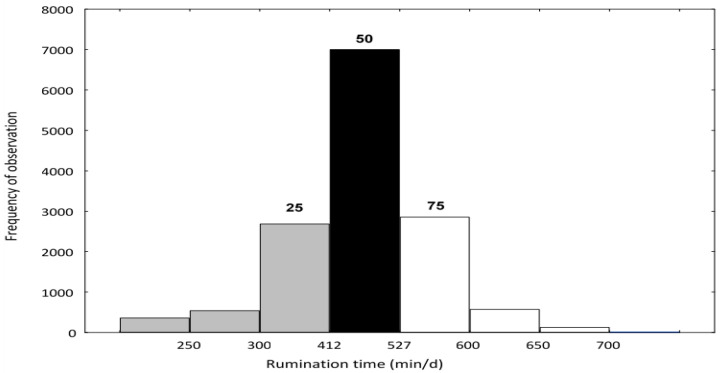
Frequency distribution of cows divided based on rumination time (min/day). Low rumination (L, grey color) to 412 min/day, medium rumination (M, black color) from 412 to 527 min/day, and high rumination (H, white color) from 527 min/day.

A total of 365 high-yielding (11,264 kg/305 days lactation) Polish Holstein-Friesian multiparous dairy cows were included in the study covering 24 to 304 days of lactation. In total, 14,274 daily complete (cow and all milk production traits) observations were collected. Data were collected in a production environment. Data from cows were assigned to three groups based on individual cow average daily rumination time (Figure 1): low rumination up to 412 min/day (L, up to the 25th rumination percentile), medium rumination from 412 to 527 min/day (M, between the 25th and 75th percentile), and high rumination above 527 min/day (H, from the 75th percentile).

The cows were milked in an automatic milking system (AMS, Astronaut, Lely Industries, NV, Maassluis, The Netherlands).

The cows received ad libitum a partial mixed ration (PMR), which was served twice a day and met requirements for 25 kg of milk yield. The animals had free and equal access to the feeding table. The cows were divided into two groups due to the management routine and not based on their characteristics. Each group had one common feeding table whose size was dependent on the number of the animals in the technological group.

The nutritional values of the feed components were calculated on the basis of the analyzed nutrient contents using NIRS (InfraXact, Foss, Hilleroed, Denmark) and the MAX^TM^ System for Dairy software (3.19, Cargill, Minneapolis, MN, USA). The diets were balanced according to the NRC (2001) system recommendations and the MAX^TM^ System for Dairy software (3.19, Cargill, Minneapolis, MN, USA).

PMR and concentrates ingredients and nutritional value are shown in Table 1.

Two concentrates (C standard and C extra) were added according to the requirements of individual cows from 0.5 to 8 kg into AMS during each milking. The proportion of C standard and C extra dispensed in AMS depended on individual milk yields and ranged from 75:25 to 70:30.

The silages were analyzed and verified two times per month using the NIRS method.

Weekly forage, concentrates, and PMR representative samples were collected, frozen, and stored (−20 °C) for further pooled monthly analyses using wet chemistry methods. On the basis of crude protein (CP, method 976.05; AOAC International, 2005), neutral detergent fiber (NDF, PN-EN ISO 16472:2007), and acid detergent fiber (ADF, PN-EN ISO 13906:2009), feeds as well as the PMR were verified. The PMR values were recalculated monthly or before a new forage from a new silo was used.

The particle size distribution of PMR samples was determined weekly by the Penn State Particle Separator system with 3 sieves (19 mm, 8 mm, 1.18 mm) and a bottom pan [18]. The mean retention of particles were: 6% (>19 mm), 48% (8–19 mm), 40.5% (1.8–8 mm), and 5.5% (<1.18 mm).

### 2.2. Rumination Time, Milk Performance, Body Weight

Rumination time was measured using electronic rumination loggers placed on the neck collars (SCR Engineers Ltd., Netanya, Israel). Loggers recorded rumination data in 2 h intervals (i.e., 12 values per day), and rumination time was expressed in minutes of rumination time recorded within each time interval. The data for rumination with accuracy (rumination mark) were read from the loggers by the readers placed in the barn connected with the Lely T4C. The daily rumination time of cow was calculated by adding 12 measurements from the day. Measurements with low accuracy (rumination mark below 98) were rejected, and all the rumination time observations of a particular cow recorded at that day were removed from the dataset (i.e., 12% of daily rumination time was set to missing).

Daily milk production, fat, and protein content were obtained from the farm management system (Lely T4C) and then used for calculating energy-corrected milk (ECM) and fat protein-corrected milk (FPCM). The ECM was calculated according to Reist et al. [19] as [(0.038 × g crude fat + 0.024 × g crude protein + 0.017 × g lactose)] × kg milk/3.14. The FPCM was calculated as [(0.337 + 0.116 × milk fat % + 0.06 × milk protein %) × kg of milk] [20].

Body weight was collected in automatic scales, and therefore, some additional filtering of the data was required. For that, data from each cow were checked separately. First, the median body weight (BW) for a cow was calculated. Second, BW values lower than 100 kg than the cow’s median BW were set to missing, as such a difference was assumed to be an erroneous record. This was confirmed by the visual inspection of the data (now shown). Third, the missing BW records were substituted by the cow’s median BW.

The AMS identified each animal during milking and saved daily information concerning body weight and milk performance.

### 2.3. Methane Measurements

The CH_4_ concentration (ppm) was measured in the air exhaled by the cows during milking in AMS using an Fourier transform infrared spectroscopy FTIR analyzer (GASMET 4030; Gasmet Technologies Oy, Helsinki, Finland) installed in the feeding bin. The samples were taken continuously, and the gas samples were analyzed every 5 s. The investigated phenotypes were daily averages obtained as described in Pszczola et al. [21]. First, the concentrations from the whole milking were averaged. Secondly, the measurements from all milkings were corrected for the diurnal variation in CH_4_. Subsequently, the corrected measurements for each cow were averaged within the day. Then, methane production was calculated in L/day following Madsen et al. [22] based on the ratios between CH_4_ and CO_2_ concentrations measured during each milking and estimated heat production.

The following average daily phenotypes were defined and analyzed: methane production (CH_4_) (L), the CH_4_ production in relation to metabolic weight (CH_4_/BW^0.75^) (L/kg), milk production (CH_4_/milk) (L/kg), energy-corrected milk (CH_4_/ECM) (L/kg), and per concentrate intake (CH_4_/concentrate intake) (L/g).

### 2.4. Statistical Analysis

Rumination time was divided into three groups according to the quartile distribution. Cows below the first quartile of rumination time were assigned to the low-L group, cows between the first and third quartile were assigned to the medium-M group, and cows above the third quartile of rumination time were placed in the high-H group.

Differences between rumination groups were assessed for each of the analyzed traits separately.

To check whether rumination time has an impact on the analyzed traits, the following model was employed:

$$y_{ijkl}=GROUP_{j}+LAC_{k}\times \sum_{n=1}^{5}\beta_{n}DIM_{ln}+cow_{i}+error_{ijkl},$$

where $y_{ijkl}$ was one of the following traits (i.e., daily rumination time, body weight, metabolic body weight, concentrate intake, concentrate intake per kg of milk, concentrate intake per metabolic weight, daily milk yield, energy-corrected milk yield, fat protein-corrected milk yield, fat, protein and lactose concentration, fat to protein ratio, daily methane production, daily methane production per metabolic weight, daily methane production per milk production, and daily methane production per concentrate intake) observed on the *i*th cow assigned to the *j*th group of rumination level (GROUP). The overall lactation curve was modeled with fourth-order Legendre polynomials separately for first, second, and further lactations. The GROUP had three levels: High, Medium, and Low. The terms **cow** and **error** were random terms.

The analyses were performed in R software [23]. The model effects were estimated using lme4 package [24], the significance of the difference between estimated marginal means was assessed using lmerTest [25] and emmeans packages [26] using Satterthwaite’s method [27] for approximating degrees of freedom enabling testing for the significance of differences between fixed effects levels. The p-values obtained for the differences between the estimated marginal means for rumination groups were adjusted using Tukey’s method for comparing 3 estimates.

## 3. Results

**Figure 2:**
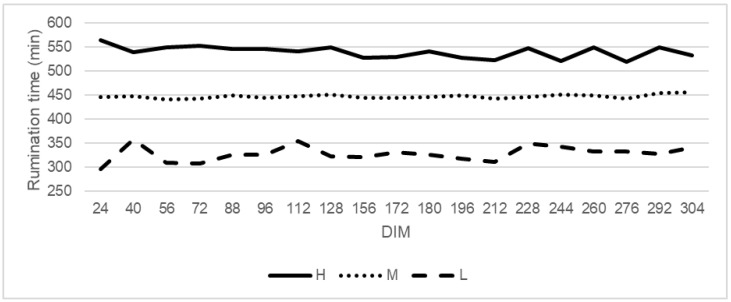
The trend of rumination time by cows classified as high (H), medium (M), and low (L) rumination animals. High rumination (H) from 527 min/day, medium rumination (M) from 412 to 527 min/day, and low rumination (L) to 412 min/day.

Differences in rumination time were observed between all the groups (H, M, and L) (Figure 2). The average daily rumination time was 195 min longer for cows in the H group in comparison to the L group and 84 min greater compared to cows, which belonged to the medium rumination time group (M) (Table 2). Mean body weight differed significantly between all the groups (H: 543 kg, M: 546 kg, L: 551 kg). The intake of concentrate from AMS was higher in low rumination cows compared to the other groups. High rumination cows were characterized by the lowest concentrate intake per their metabolic body weight (BW^0.75^, 39.58 g/kg) and differed both from medium rumination (40.34 g/kg) and low rumination cows (40.32 g/kg). Rumination time had no effect on concentrate intake on milk yield.

Rumination time had no effect on milk, energy-corrected milk, as well as fat and protein-corrected milk yield (Table 3). High rumination cows had an effect on lower fat concentration in milk (3.75%) compared with the M and L groups (3.94% and 3.80%, respectively). Differences between rumination time groups on protein and lactose concentrations in milk were not confirmed. The fat and protein ratio was lower in high rumination cows (1.14) compared to the low (1.15) and medium (1.15) rumination cows. Rumination time had no effect on the number of milkings in AMS, which were on average 2.85 per day.

The significant effect of rumination time on methane (CH_4_) emission was observed in all the groups. The highest daily CH_4_ production was noted in low rumination cows (412.47 L), which emitted 1.8% more CH_4_ than medium rumination cows (404.99 L) and 4.2% more than low rumination cows (395.80 L). The cows from the high rumination group had a lower daily CH_4_ production per BW^0.75^ (3.59 L/kg) compared to both groups, medium rumination cows (3.67 L/kg) and low rumination cows (3.68 L/kg).

Rumination time had a positive effect on daily methane production per kg of milk. Cows from the high rumination group produced less daily CH_4_ per kg of milk (11.52 L/kg) compared to medium (11.86 L/kg) and low (12.07 L/kg) rumination cows. Similar observations were noted for daily CH_4_ production per ECM unit (11.79 L/kg, 12.07 L/kg, 12.26 L/kg). Daily lower methane production per concentrate intake unit was highest in medium rumination cows (0.12 L/g) compared to the L group (0.10 L/g).

**Figure 3:**
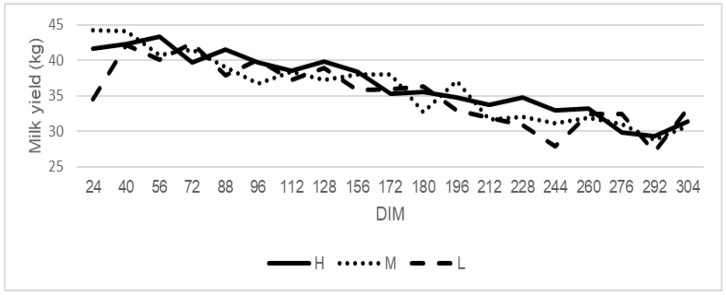
The trend for milk production in cows classified as high (H), medium (M), and low (L) rumination animals. High rumination (H) from 527 min/day, medium rumination (M) from 412 to 527 min/day, and low rumination (L) to 412 min/day.

**Figure 4:**
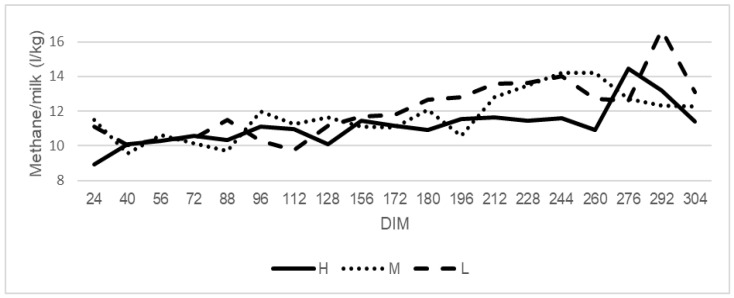
The trend for methane emission on milk production (CH4/milk) in cows classified as high (H), medium (M), and low (L) rumination animals. High rumination (H) from 527 min/day, medium rumination (M) from 412 to 527 min/day, and low rumination (L) to 412 min/day.

Daily methane yield (kg) was higher at the beginning of the lactation and decreased toward the end of the milking period (Figure 3), whereas the methane production per kg of milk was low at the beginning of the lactation and increased toward the end of the lactation (Figure 4).

## 4. Discussion

We hypothesized that a longer rumination time would be connected with lower daily methane production per milk unit in high-yielding dairy cows fed a partial mixed ration based on maize silage without pasture access. Cows from all the groups (H, M, and L) ruminated approximately 458 min per day, which is in the range reported in the literature by White et al. [28], who analyzed 179 cows with a mean rumination time of 436 min/day, ranging from 236 to 610 min/day, as well as Zetouni et al. [16], who recorded 443 min/day as average rumination time during Danish Holstein cows lactation. Cows from the high rumination group ruminated 551 min/day, which was an 84 min increase compared to the medium (467 min/day) and 195 min more compared to the low ruminating cows (356 min/day). Similar differences of rumination time in grazing cows were reported by Watt et al. [10].

Rumination time had no effect on milk, energy-corrected milk as well as fat and protein-corrected milk production. Despite a positive relationship between rumination time and milk production in early lactation [29] and mid-lactation [4], which has been reported earlier, Stone et al. [30] noted a weak correlation between both phenotypes (*r* = 0.30). The positive relationship between rumination time and milk production may be indirectly related to dry matter intake. Nevertheless, dry matter intake may indirectly cause a positive relationship between rumination time and milk yield, and the association between dry matter intake and rumination time can also depend on diet composition [3].

Moreover, Stone et al. [30] explained that the reason for the different results shown by various authors was due to differences in methods of rumination activity detection. In an early study, rumination was estimated based on direct visual observations, and results can be different when measured by an automated rumination logging system [30].

Watt et al. [10] observed a positive association with rumination time and greater milk production, concentrate intake from AMS, as well as estimated dry matter intake by grazing cows. It is commonly known that the main factors of rumination time are connected with the chemical and physical characteristics of the diets, but according to Beauchemin et al. [3], who described a positive relationship between rumination time and dry mater intake in dairy cows, on this basis, we can assume that high rumination cows were also fed a more PMR-based diet. Schirmann et al. [31] showed that cows that ruminated more time per day spent less time feeding (*r* = −0.34), and rumination times did not relate to dry matter intake (*r* = 0.11). In the present study, differences in concentrate intake across the groups were not detected. Rumination time had a slight effect on milk composition; the cows that ruminated longer (H) had only less fat concentration without differences in protein and lactose concentrations in milk. Similarly, a negative correlation between rumination time and milk fat concentration during the first month of lactation in cows older than the third lactation was noted by Kaufman et al. [9]. It would appear that an increase in rumination time should be directly connected with better rumen homeostasis and fiber microbial degradation and an increase in fat percentage [32]. Less milk fat concentration in high ruminating cows (H) may be connected with their higher milk yield, while it may also be a consequence of the enhanced availability of glucose for the synthesis of lactose in milk without any increase in volatile fatty acids or long-chain fatty acids for butterfat synthesis. Rumination time had no effect on protein concentration in milk, which is in agreement with the observations reported by Kaufman et al. [9], who found no association between milk protein and rumination time in dairy cows during the first month of lactation. Different results, i.e., a negative relationship between rumination time and milk production, protein content in milk, but a positively association with milk fat concentration in a study of mid-lactation Holstein and Swedish Red cows were reported by Byskov et al. [33].

Rumination time had a positive effect on a decrease in methane production; cows assigned to the high ruminating group produced less methane than medium and lower ruminating groups, and medium ruminating cows produced less methane than cows with a lower daily rumination time. A similar result, negative genetic correlation between methane and rumination time was estimated by López-Paredes et al. [15], who collected methane data from 14 to 21-day periods. This results are different from those of Zatouni et al. [16], who observed a lack of relationship between rumination time and methane emission by high-yielding dairy cows. Phenotypes, methane emission, and rumination activity are affected by many factors that are hard to be accounted for, and therefore, the results from other studies can differ. Additionally, it is known that increasing NDF from forages in the dairy cows diets stimulates rumination activity, increases saliva production, and via buffering rumen fluid increases the production of acetate in the rumen, leading to higher methane production [33]. On the other hand, decreasing NDF from forage and an increase in concentrates intake may be associated with a decreased rumen pH, leading to an increase in the levels of propionate and a decrease in acetate and butyrate levels while decreasing hydrogen equivalents that would be converted to methane and are inhibitors in methanogenesis. Different results from current study, higher methane emissions from high ruminating grazing cows were shown by Watt et al. [10]. An explanation of these differences may be attributed to the different body weights of high and low ruminating cows in both experiments. In a study described by Watt et al. [10], high ruminating grazing cows were heavier than low ruminating grazing cows in contrast to the present study, where high ruminating cows had lower body weight. Additionally, the high ruminating cows had lower methane emissions per metabolic body weight than cows that spent less time on rumination. Lower methane production in high ruminating cows per body weight may be connected with lower body weight as well as lower methane production by cows from this group.

In the present study, high ruminating cows had a lower daily methane production per milk unit as well as energy-corrected milk than other cows, which spent less time on rumination. A reduction of methane emission per milk production in high ruminating cows with similar milk yield between the three groups is connected only with the lowest methane emission. A reduction of methane production per unit of product was also observed in high ruminating grazing dairy cows [10]. Knapp et al. [14] described that diets containing more energy or with better digestibility increase net energy intake, and when this energy is partitioned into milk production, a decrease in methane emission per ECM yield can be observed. In addition, Aguerre et al. [34] observed a decrease in methane per ECM production when non-fiber carbohydrates were elevated through an increase in concentrate intake from 32 to 53% in the diet.

We collected 14,274 records of daily methane emissions recorded throughout lactation from 24 to 304 days to obtain high reliability of the daily methane production estimates. Including individual dry matter intake levels would provide additional insights; however, they was not possible to collect due to the very large number of collected observations and technical difficulties. Methane emission measurements are highly variable between animals and within the lactation period. Thus, studies on methane emission should be conducted on a large number of animals and cover a long time period and the association of rumination time that best indicates the physiological state of ruminal fermentation at optimal levels to ensure animal welfare and health.

## 5. Conclusions

In conclusion, the results confirmed the hypothesis that a longer rumination time is related to lower methane emission per milk unit in high-yielding dairy cows fed a partial mixed ration based on maize silage without pasture access.

## Figures and Tables

**Table 1:** Average ingredients and nutrient composition of the experimental partial mixed ration diet (PMR) and concentrates (C standard, C extra).

Items	
PMR	
Ingredients, g/kg DM	
maize silage	337
alfalfa silage	149
wheat straw	91
wheat grain	81
sugar beet pulp silage	81
brewer’s grain silage	76
maize grain silage	69
rapeseed meal	61
ProStim Soy balance	18
minerals, vitamins, and feed additives	37
Nutritional value, in kg DM	
NEL_3x_	1.49 Mcal/kg
CP	150 g/kg
RUP	35%
NDF	356 g/kg
ADF	223 g/kg
NFC	370 g/kg
**C standard**	
Nutritional value, in kg DM	
NEL_3x_	1.73 Mcal/kg
CP	220 g/kg
**C extra**	
Nutritional value, in kg DM	
NEL_3x_	1.99 Mcal/kg
CP	267 g/kg

DM—dry matter; NEL—Net Energy Lactation; CP—crude protein; RUP—rumen undegraded protein; NDF—neutral detergent fiber; ADF—acid detergent fiber; NFC—non-fiber carbohydrates; ProStim Soy balance—soybean meal, brewer’s grain, urea, 1.79 Mcal NEL_3x_, CP 784 g/kg DM; minerals, vitamins, and feed additives—sodium 12.5%, calcium 13.2%, phosphorus 2.5%, vit. A 220,000 IU, vit. D_3_ 50,000 IU, vit. E 850 mg/kg, niacin 7800 mg/kg, vit. B_12_ 700 µg/kg, biotin 21,000 µg/kg, folic acid 20 mg/kg, magnesium 64 g/kg, iron 310 mg/kg, manganese 2000 mg/kg, copper 500 mg/kg, zinc 2340 mg/kg, iodine 44 mg/kg, cobalt 8.8 mg/kg, selenium 10 mg/kg, copper organic 220 mg/kg, manganese organic 440 mg/kg, zinc organic 1320 mg/kg, selenium organic 2.5 mg/kg, vit. E total 1800 mg/kg, proviox eqw vit. E 1800 mg/kg, mycofix plus 18 g/kg, Diamond XP LS 20,000 mg/kg, klinoptylolit 82,976 mg/kg, bentonite 5400 mg/kg, *S. cerevisiae* 70,000,001,024 cfu/kg; both concentrates based on soybean meal, rapeseed meal, corn, wheat and barley grains, barley germ.

**Table 2:** Estimated marginal means for rumination time, body weight, and concentrate intake of low (L), medium (M), and high (H) rumination cows.

Variable	Rumination Group			SD
	L	M	H	
No. observations	3650	7029	3595	N.A.
Rumination time (min/day)	356.39 ^a^	467.30 ^b^	551.06 ^c^	94.81
BW (kg)	551.46 ^a^	545.80 ^b^	543.44 ^c^	85.87
BW^0.75^ (kg)	113.21 ^a^	112.35 ^b^	112.01 ^b^	13.39
Concentrate intake (g/day)	4360 ^a^	4264 ^b^	4239 ^b^	1368
Concentrate intake/milk yield (g/kg)	123.73	121.65	120.97	53.12
Concentrate intake BW^0.75^ (g/kg)	40.32 ^a^	40.34 ^a^	39.58 ^b^	11.99

^a–c^ Estimated marginal means within a row with different superscripts differ significantly (*p* < 0.05); Low rumination (L) to 412 min/day, medium rumination (M) from 412 to 527 min/day, and high rumination (H) from 527 min/day; SD—overall standard deviation.

**Table 3:** Estimated marginal means for milk and methane production phenotypes of low (L), medium (M), and high (H) rumination cows.

Phenotypes	Rumination Groups			SD
	L	M	H	
**Milk production, composition and AMS use**				
Milk (kg/day)	35.46	35.28	35.26	7.84
ECM (kg/day)	34.42	34.31	34.17	6.88
FPCM (kg/day)	33.95	33.83	33.70	6.81
Fat (%)	3.80 ^a^	3.94 ^a^	3.75 ^b^	0.55
Protein (%)	3.29	3.30	3.29	0.18
Lactose (%)	4.97	4.97	4.97	0.11
Fat: protein	1.15 ^a^	1.15 ^a^	1.14 ^b^	0.17
Milkings/day	2.84	2.84	2.86	0.81
**Daily methane production**				
CH_4_ (L)	412.47 ^a^	404.99 ^b^	395.80 ^c^	87.16
CH_4_/BW^0.75^ (L/kg)	3.68 ^a^	3.67 ^a^	3.59 ^b^	0.78
CH_4_/milk (L/kg)	12.07 ^a^	11.86 ^b^	11.52 ^c^	3.49
CH_4_/ECM (L/kg)	12.26 ^a^	12.07 ^b^	11.79 ^c^	3.29
CH_4_/concentrate intake (L/g)	0.10 ^a^	0.12 ^b^	0.11 ^ab^	0.15

^a–c^ Estimated marginal means within a row with different superscripts differ (*p* < 0.05); low rumination (L) to 412 min/day, medium rumination (M) from 412 to 527 min/day, and high rumination (H) from 527 min/day; SD—overall standard deviation.

## Data Availability

Data is available at a reasonable request to the corresponding authors.
